# Cell Culture and Electron Microscopy for Identifying Viruses in Diseases of Unknown Cause

**DOI:** 10.3201/eid1906.130173

**Published:** 2013-06

**Authors:** Cynthia S. Goldsmith, Thomas G. Ksiazek, Pierre E. Rollin, James A. Comer, William L. Nicholson, Teresa C.T. Peret, Dean D. Erdman, William J. Bellini, Brian H. Harcourt, Paul A. Rota, Julu Bhatnagar, Michael D. Bowen, Bobbie R. Erickson, Laura K. McMullan, Stuart T. Nichol, Wun-Ju Shieh, Christopher D. Paddock, Sherif R. Zaki

**Affiliations:** Centers for Disease Control and Prevention, Atlanta, Georgia, USA (C.S. Goldsmith, P.E. Rollin, J.A. Comer, W.L. Nicholson, T.C.T. Peret, D.D. Erdman, W.J. Bellini, B.H. Harcourt, P.A. Rota, J. Bhatnagar, M.D. Bowen, B.R. Erickson, L.K. McMullan, S.T. Nichol, W.-J. Shieh, C.D. Paddock, S.R. Zaki);; University of Texas Medical Branch, Galveston, Texas, USA (T.G. Ksiazek)

**Keywords:** Viruses, electron microscopy, cell culture, emerging diseases, SARS coronavirus, Nipah virus, lymphocytic choriomeningitis virus, West Nile virus, Cache Valley virus, Heartland virus

## Abstract

During outbreaks of infectious diseases or in cases of severely ill patients, it is imperative to identify the causative agent. This report describes several events in which virus isolation and identification by electron microscopy were critical to initial recognition of the etiologic agent, which was further analyzed by additional laboratory diagnostic assays. Examples include severe acute respiratory syndrome coronavirus, and Nipah, lymphocytic choriomeningitis, West Nile, Cache Valley, and Heartland viruses. These cases illustrate the importance of the techniques of cell culture and electron microscopy in pathogen identification and recognition of emerging diseases.

Thin section and negative stain electron microscopy (EM) examination of viruses grown in cultured cells have been instrumental in determining an etiologic agent in numerous disease outbreaks caused by previously unknown viruses. Many examples have been reported. In 1976, EM of cell culture isolates identified the causative virus of an outbreak of hemorrhagic fever in Zaire as a member of the family *Filoviridae*, now known as Zaire ebolavirus (*1*–*3*). Reston ebolavirus was another previously unrecognized virus that was detected by cell culture and EM in 1989; it was isolated from cynomolgus monkeys imported into the United States from the Philippines (*4*). In Australia in 1994, during an outbreak of fatal respiratory disease in horses and influenza-like illness in humans, a previously unknown virus, Hendra virus, was isolated in culture and recognized as a member of the family *Paramyxoviridae* by EM (*5*,*6*). An outbreak of an unidentified rash illness in humans, associated with sick prairie dogs, occurred in the upper midwestern United States in 2003, and EM detected a poxvirus from a cell culture isolate, which was later characterized as monkeypox virus (*7*,*8*). Recently, the etiologic agent of severe fever with thrombocytopenia syndrome in China was isolated and identified by EM as a member of the family *Bunyaviridae* (*9*).

Inoculation of patient specimens onto cultured cells or into laboratory animals enables biologic amplification of virus particles to levels where they can be detected by EM and identified to a virus family because, with a few exceptions (*10*), the morphologic features of all viruses within a given family are the same. Once recognized by EM, the findings can be confirmed by other techniques, including serologic testing, immunohistochemical (IHC) and indirect fluorescence antibody (IFA) assays, and molecular methods that can further characterize the virus to species and strain.

Cell culture methods are relatively unbiased, restricted only by the ability of the virus to grow in a particular cell line. Vero E6 cells, considered one of the most permissive of all cell lines, provide an extremely versatile medium for recovery of unknown pathogens. EM is also an unbiased assay in that there is no need for specific immunologic probes, and has the added advantage of being able to detect and classify the unknown agent. EM observations of cell culture isolates can provide the first clue of an etiologic agent and guide subsequent laboratory and epidemiologic investigations. Detection of a pathogen is critical during outbreaks because identification of an etiologic agent enables public health officials to mount a timely response and limit further spread of the agent involved. In addition, pathogen identification is invaluable in individual cases of severe illness in which an infection is caused by an undetermined agent. We report several cases where cell culture and EM at the Centers for Disease Control and Prevention (CDC) enabled initial recognition and identification of a cause of the viral illness.

## Tissue Culture

Of the variety of tissue culture cells available, many are maintained in minimal essential medium at 37°C. Once cells have become confluent, they can be inoculated with suspensions of ground tissues (e.g., lung, liver), incubated for 1 hour, and grown until there is an ≈3+ cytopathic effect. Cells are then removed from the growth vessel by scraping or with glass beads, rinsed with 0.1 mol/L phosphate buffer, centrifuged, and fixed in buffered 2.5% glutaraldehyde for 1 hour.

## Electron Microscopy

Specimens are postfixed in 1% osmium tetroxide, en bloc stained with 4% uranyl acetate, dehydrated through a graded series of alcohol and propylene oxide, and embedded in a mixture of Epon substitute and Araldite. Thin sections are stained with 4% uranyl acetate and Reynolds’s lead citrate.

## Family *Coronaviridae*

Beginning in late 2002, an outbreak of severe pneumonia associated with human deaths occurred in Guangdong Province, China, which escalated to a global pandemic of respiratory illness in early 2003. The World Health Organization reported 8,098 probable cases in 29 countries, and the disease killed 774 persons worldwide (*11*). Patients had an influenza-like illness with fever, cough, dyspnea, headache, and consolidation shown on chest radiographs, and the disease became known as severe acute respiratory syndrome (SARS). Isolation of a virus was achieved in several laboratories around the world by inoculating respiratory specimens onto cell culture, and thin section EM first identified the isolate as a coronavirus (*12*). This finding was quickly corroborated by negative stain EM, IHC assay, serologic testing, and molecular assays. Thus, once the isolate was identified as a coronavirus by EM, the focus of the laboratory investigation shifted toward verification of this finding. The natural reservoir for the progenitor of SARS coronavirus is most likely the Chinese horseshoe bat (*Rhinolophus sinicus*) because SARS corona-like viruses were identified and characterized in these animals (*13*,*14*).

**Figure 1:**
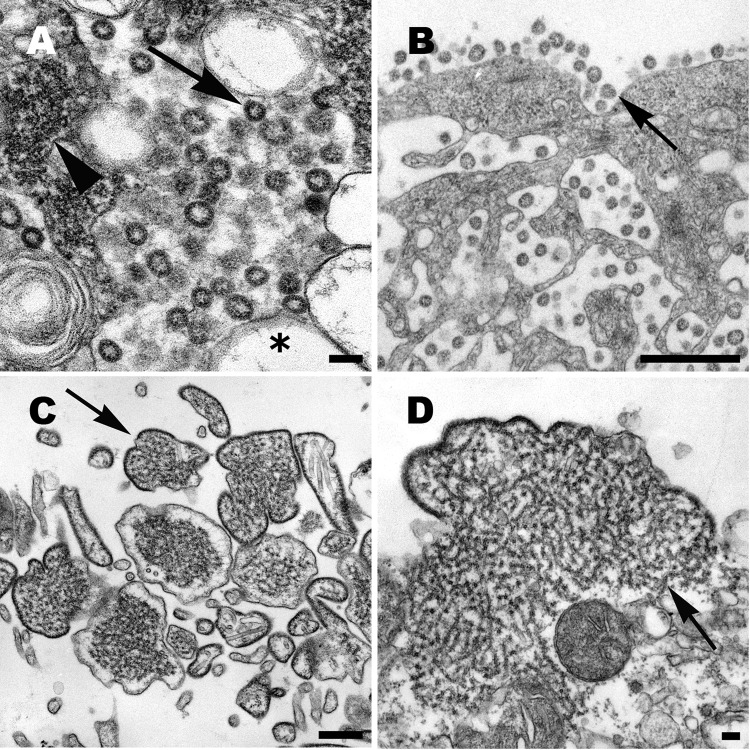
A) Cell culture isolate of severe acute respiratory syndrome coronavirus, in which virions are seen in the cisternae of the budding compartment (arrow). Also present are an inclusion of viral nucleocapsids (arrowhead) and double-membrane vesicles (asterisk). Scale bar = 100 nm. B) Coronavirus particles in cytoplasmic vesicles that appear to migrate to the cell surface. Virions are seen lining the cell membrane (arrow), a characteristic feature of this virus. Scale bar = 500 nm. C) Large, pleomorphic, extracellular Nipah virus particles (arrow), in which the viral envelope encloses the nucleocapsids. Scale bar = 500 nm. D) Nipah virus nucleocapsids (arrow) aggregate in the cytoplasm and become tightly apposed to the cell membrane as the virus begins the process of budding. Scale bar = 100 nm.

Coronavirus particles are mostly spherical, sometimes pleomorphic, and have an average diameter of ≈80 nm (Figure 1, panel A). Nascent particles are formed when the helical nucleocapsids align along cytoplasmic membranes of the budding compartment (the membrane region between the rough endoplasmic reticulum and the Golgi complex), and virions obtain their membranes by budding into the cisternal lumen. Also found in some infected cells are accumulations of the viral nucleocapsids, and double-membrane vesicles that are believed to be the replication complex for this virus. Particles accumulate in cytoplasmic vesicles, which migrate toward the cell surface and fuse with the cell membrane, releasing the virus particles. Many virions remain adsorbed to the cell membrane, which gives infected cells a characteristic appearance of an outer layer of particles (Figure 1, panel B) (*15*).

## Family *Paramyxoviridae*

In Peninsular Malaysia and Singapore during 1998–1999, an outbreak of viral encephalitis with a high mortality rate occurred among men who had been exposed to pigs. The illness was characterized by fever and headache, followed by a rapid deterioration in consciousness; >100 deaths were reported. Concurrently, respiratory illness increased among pigs in the same region. A virus was isolated in Malaysia from cerebrospinal fluid of a patient and was identified by EM as a member of the family *Paramyxoviridae* (*16*,*17*). It was shown to be the etiologic agent for human and swine diseases and is now known as Nipah virus. Serologic and PCR findings for the isolate indicated that Nipah virus was closely related to Hendra virus, a novel paramyxovirus which had been isolated in Australia in 1994 (*5*). The natural reservoir for Nipah virus was found to be flying foxes (*Pteropus hypomelanus* and *P. vampyrus*) (*18*).

Nipah virus particles are pleomorphic and vary greatly in size. Particles are composed of a tangle of nucleocapsids enclosed within the viral envelope, which contains surface projections 12 nm in length (Figure 1, panel C). Negative stain EM showed that the helical nucleocapsids have a herringbone appearance and an average diameter of 21 nm. The nucleocapsids can aggregate in the cytoplasm to form inclusions or migrate to the cell surface where they become tightly apposed with the cell membrane as the virus buds. (Figure 1, panel D) (*19*).

## Family *Arenaviridae*

Organ and tissue transplantation have become relatively common surgical procedures, and on rare occasions, transplant recipients can become infected when a pathogen is transmitted from the donor. The immunocompromised status of the organ recipients enables amplification of the pathogen, which may lead to illness and death. In recent years, there have been several unexpected donor-derived clusters of infection, including reports of transmission of rabies virus, West Nile virus, and *Trypanosoma cruzi*, the etiologic agent of Chagas disease (*20*–*23*). In 2 clusters of organ transplantation, 1 in 2003 and 1 in 2005, symptoms such as unexplained fever, graft dysfunction, and altered mental status developed in recipients; 7 of the 8 recipients died. An etiologic agent was isolated from cerebrospinal fluid of a patient in the 2003 cluster, and identified by EM as belonging to the family *Arenaviridae*. IFA assay and PCR showed that the agent was lymphocytic choriomeningitis virus (LCMV), an arenavirus transmitted by rodents (*24*). In immunocompetent humans, this virus typically causes a subclinical infection that rarely results in death.

**Figure 2:**
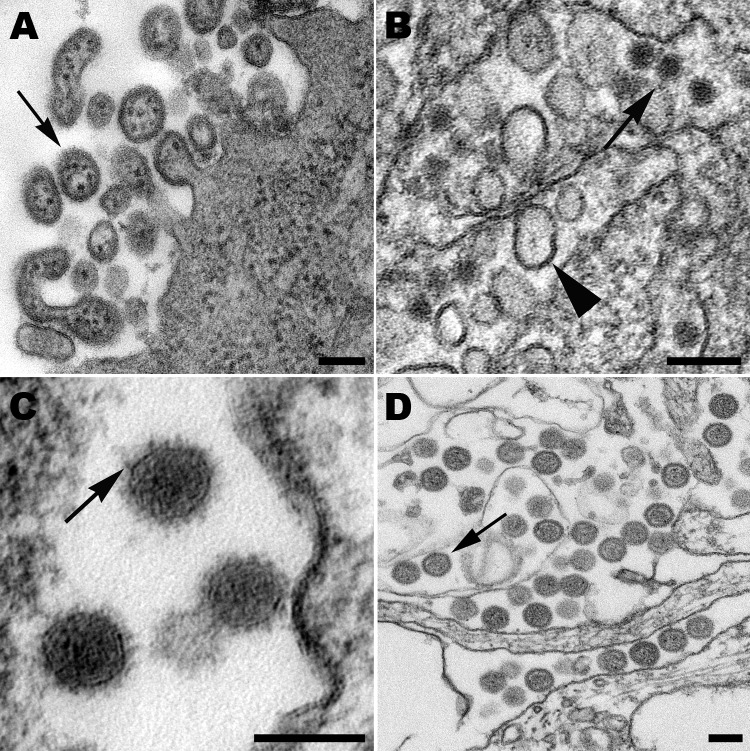
A) Extracellular lymphocytic choriomeningitis virus particles (arrow) containing cellular ribosomes. B) West Nile virus particles (arrow) in the lumen of the rough endoplasmic reticulum of an infected cell. Also in the cisternae are smooth membrane vesicles (arrowhead). C) High magnification of Cache Valley virus particles within a Golgi vesicle, showing small surface projections (arrow). D) Extracellular, spherical Homeland virus particles (arrow) with a slightly granular core. Scale bars = 100 nm.

Arenaviruses are mostly spherical, although there can be pleomorphic forms (Figure 2, panel A). Most notable is the inclusion of ribosomes inside the virus particles. Virions have a mean diameter of 110–130 nm but can vary in size. The virus particles bud at the cell membrane and have a dense outer envelope with small surface projections, and the appearance of the interior of the particles ranges from slightly granular to lucent.

## Family *Flaviviridae*

Cell culture isolation and EM examination were also instrumental in the diagnosis of an etiologic agent in a patient with an unusual clinical manifestation. A 59-year-old man in Florida, USA, had hemorrhagic symptoms, including fever, hypotension, rash, loose bloody stools, and acute renal failure; he died within 1 week. The patient reported having recent arthropod bites, and a rickettsial disease was suspected. A serum sample was obtained from the patient, but it was negative by IFA assay for several rickettsial agents. A punch biopsy specimen of a skin lesion was obtained and homogenized and inoculated onto cell culture. A cytopathic effect was subsequently noted, the culture was examined by EM, and the isolate was recognized as belonging to the family *Flaviviridae*. PCR showed that the isolate was West Nile virus (*25*), which is only rarely known to cause a hemorrhagic-like disease.

Flavivirus-infected cells show a proliferation of the endoplasmic reticulum membranes with viral particles found within the lumen. Virions are ≈40 nm in diameter and have a dense core of 25–30 nm (Figure 2, panel B). Surrounding the core is an electron-lucent halo enclosed by the viral envelope. Smooth membrane structures having a mostly clear interior with a slight web-like arrangement are also found in the lumen of the endoplasmic reticulum and have been shown to be the replication complexes for this virus (*26*). Accumulations of dense convoluted membranes are also found in infected cells (*27*).

## Family *Bunyaviridae*

There were 2 instances at CDC in which a bunyavirus was isolated from patients and identified by EM. In the first instance, a 28-year-old man in North Carolina, USA, had myalgias, fever, chills, and headache; his illness progressed to severe encephalitis and multiorgan failure, and resulted in death. An isolate was obtained at Duke University Medical Center (Durham, NC, USA) and at CDC from blood, serum, and cerebrospinal fluid, and EM examination recognized a virus belonging to the family *Bunyaviridae*. The virus was found to belong to the Bunyamwera serogroup (genus *Orthobunyavirus*) by ELISA and identified as Cache Valley virus by PCR and sequencing (*28*). Cache Valley virus was originally isolated from mosquitos in 1956, and the patient reported receiving many mosquito bites 2 weeks before the onset of illness. This finding was the first reported isolation of Cache Valley virus from a human case-patient.

In a recent second instance, a bunyavirus was isolated from 2 patients in northwestern Missouri, USA, who had a history of tick bites. Both patients had fever, fatigue, diarrhea, thrombocytopenia, and leukopenia, but recovered from their illnesses and were released after 10–12 days of hospitalization. An *Ehrlichia* sp. was initially suspected, and leukocytes from the patients were inoculated onto DH82 cells, a canine monocyte cell line. When cytologic changes were seen in the culture, cells were processed for EM examination. Rather than a bacteria, a bunyavirus was recognized. It was identified as a new member of the genus *Phlebovirus* by deep sequencing and is now known as Heartland virus (*29*).

Virus particles in bunyavirus-infected cells bud upon smooth cytoplasmic membranes and are found in vesicles and also extracellularly (Figure 2, panels C, D). The spherical, enveloped virions have surface projections visible on the surface of some particles. Virions have a granular interior with varying densities.

## Conclusions

For decades, the combination of the classical techniques of virus isolation in tissue culture and examination by EM has been critical in detection of previously unrecognized viruses. Cell culture is a fundamental procedure that can be accomplished in most hospital microbiology laboratories and should be considered if an infectious viral agent is suspected. Although some examples provided in this report were handled in biosafety level 3 laboratories at CDC, other examples were handled as routine microbiological isolations in hospitals or public health departments equipped to perform routine virus isolation.

Other useful laboratory methods for diagnosis of an unknown virus include serologic testing; IFA, histopathologic, and IHC assays; PCR; and sequencing. Metagenomics with deep sequencing is a recent advancement of a molecular technique that enables genomic analysis of microorganisms without the need to isolate and culture pathogens. High-throughput sequencing uses random amplified DNA products and compares the obtained product sequences with available extensive banks of sequences for final identification of the agent detected. Because random primers are used to nonspecifically amplify templates for sequencing, there is no need for prior knowledge of the suspected target. This technology is advancing rapidly and improvements in the field will undoubtedly solidify its use in the field of pathogen discovery. Previously unknown viruses that have been recognized by using this technique include Schmallenberg virus (*30*), Lloviu virus (*31*), and Bas-Congo virus (*32*).

As molecular diagnostic techniques progress in scope and magnitude, it is critical to retain and use classical techniques, including cell culture and EM, that complement the advances in molecular methods. There is a continuing need to train younger scientists in these traditional methods to maintain an underlying expertise. In particular, with EM, the electron microscopist needs to be able to differentiate between infectious agents and artifacts or look-alike structures. The combination of cell culture and EM is an unbiased approach to identification of a previously unrecognized pathogen. Similarly, unbiased thinking and collaboration among clinicians, epidemiologists, microbiologists, and laboratorians who use different technologies are critical for successful investigations of diseases of unknown origin.
